# Brain experiments imply adaptation mechanisms which outperform common AI learning algorithms

**DOI:** 10.1038/s41598-020-63755-5

**Published:** 2020-04-23

**Authors:** Shira Sardi, Roni Vardi, Yuval Meir, Yael Tugendhaft, Shiri Hodassman, Amir Goldental, Ido Kanter

**Affiliations:** 1 Department of Physics, Bar-Ilan University, Ramat-Gan, 52900 Israel; 2 Gonda Interdisciplinary Brain Research Center and the Goodman Faculty of Life Sciences, Bar-Ilan University, Ramat-Gan, 52900 Israel

**Keywords:** Biological physics, Complex networks

## Abstract

Attempting to imitate the brain’s functionalities, researchers have bridged between neuroscience and artificial intelligence for decades; however, experimental neuroscience has not directly advanced the field of machine learning (ML). Here, using neuronal cultures, we demonstrate that increased training frequency accelerates the neuronal adaptation processes. This mechanism was implemented on artificial neural networks, where a local learning step-size increases for coherent consecutive learning steps, and tested on a simple dataset of handwritten digits, MNIST. Based on our on-line learning results with a few handwriting examples, success rates for brain-inspired algorithms substantially outperform the commonly used ML algorithms. We speculate this emerging bridge from slow brain function to ML will promote ultrafast decision making under limited examples, which is the reality in many aspects of human activity, robotic control, and network optimization.

## Introduction

Machine learning is based on Donald Hebb’s pioneering work; seventy years ago, he suggested that learning occurs in the brain through synaptic (link) strength modifications^1^. A synaptic strength modification typically lasts tens of minutes^2^ while the clock speed of a neuron (node) ranges around one second^3^. Although the brain is comparatively slow, its computational capabilities outperform typical state-of-the-art artificial intelligence algorithms. Following this speed/capability paradox, we experimentally derive accelerated learning mechanisms based on small datasets, where their utilization on gigahertz processors is expected to lead to ultrafast decision making.

Unlike modern computers, a well-defined global clock does not govern brain dynamics; instead, they are a function of relative event timing (e.g., stimulations and evoked spikes)^4^. According to neuronal computational, using decaying input summation via its ramified dendritic trees, each neuron sums the asynchronous incoming electrical signals and generates a short electrical pulse (spike) when its threshold is reached. For each neuron, synaptic strength is slowly modified based on the relative timing of inputs from other synapses; if a signal is induced from a synapse without generating a spike, its associated strength is modified based on the relative timing to adjacent spikes from other synapses on the same neuron^5^.

Recently it was experimentally demonstrated that each neuron functions as a collection of independent threshold units^6^. After signals arrive via one of the dendritic trees, each threshold unit is activated. Additionally, a new type of adaptive rule was experimentally observed based on dendritic signal arrival timing^7^, which is similar to the slow adaptation mechanism currently attributed to synapses (links). This dendritic adaptation occurs on a faster timescale: it requires approximately five minutes, while synaptic modification requires tens of minutes or more.

## Results

**Figure 1:**
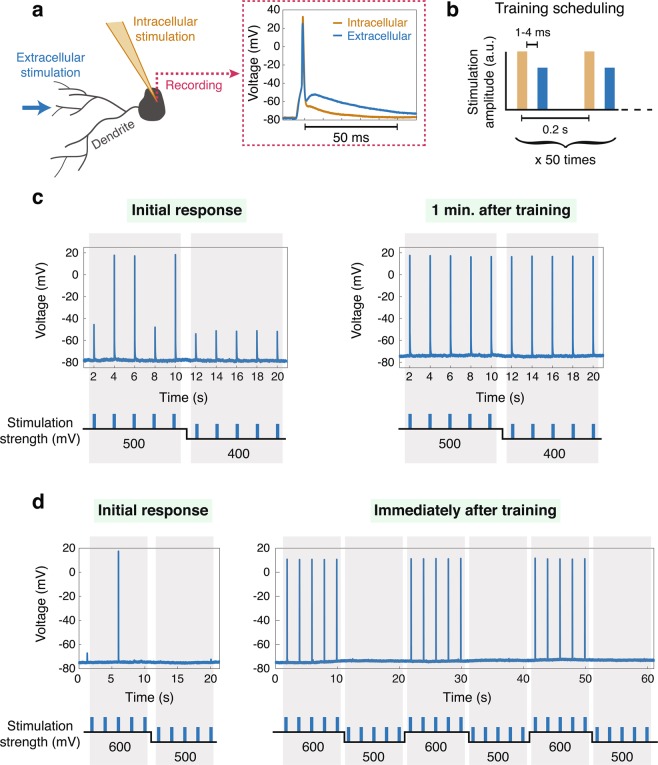
Experimental results indicate that adaptation rates increase with training frequency. (**a**) The experimental scheme where a patched neuron is stimulated intracellularly via its dendrites (Materials and Methods) and a different spike waveform is generated for each stimulated route. (**b**) The scheduling for coherent training consists of repeated pairs of intracellular stimulations (orange) generating a spike followed by an extracellular stimulation (blue) with the lack of a spike. (**c**) An example of the first type of experiments, where decreasing extracellular stimulation amplitude is used to estimate the threshold using intracellular recording (left), and enhanced responses measured a minute after the termination of the training, **b** (right). (**d**) An example of the second type of experiment, similar to **c**, where enhanced responses are observed 10 seconds after the termination of the training (Materials and Methods).

In this study, dendritic adaptation was experimentally examined at a higher stimulation frequency, 5 Hz, using the training pattern of previous experiments run at 0.5 to 1 Hz. We planted neuronal cultures on a multi-electrode-array with added synaptic blockers, which extracellularly stimulated a patched neuron via its dendrites (Fig. 1a and Materials and Methods). The adaptation process consisted of a training set: 50 pairs of stimulations. After an above-threshold intracellular stimulation, an extracellular stimulation that did not evoke a spike arrived with a predefined delay, typically 1 to 4 ms (Fig. 1b). We primarily take into account differing extra- and intra-spike waveforms (Fig. 1a, right), which presumably activated the neuron from two independent dendritic trees^7^.

By comparing the amplitudes of intracellular responses and extracellular stimulation before and after the training procedure, we quantified the effect of the neuronal adaptation. To quantify the initial response, the extracellular stimulation amplitude was decreased until no reliable evoked spikes were observed (Fig. 1c,d, left).

In the first type of experiment, one minute after the training terminated, we measured the enhanced responses and witnessed dendritic adaptation (Fig. 1c, right). When compared with the visible adaptation time for the 1 Hz training, that of the 5 Hz training was substantially faster. Occasionally, the visible effect of adaptation was further enhanced after more time passed (Supplementary Fig. 1), suggesting by extrapolation that adaptation might occur in much less than one minute. Because after training, the initialization and compilation of subsequent experiments required a minimal time lag (one minute), the feasibility of such ultrafast adaptation was impossible to examine.

To overcome this limitation, we introduced a second type of experiment (Fig. 1d): shortly after the end of the training procedure, the neuron was extracellularly stimulated using two predefined amplitudes with unreliable responses. Without requiring any new experimental methods or compilations (Materials and Methods), this procedure pinpointed dendritic adaptation within only 10 seconds of the training termination (Fig. 1d, right).

**Figure 2:**
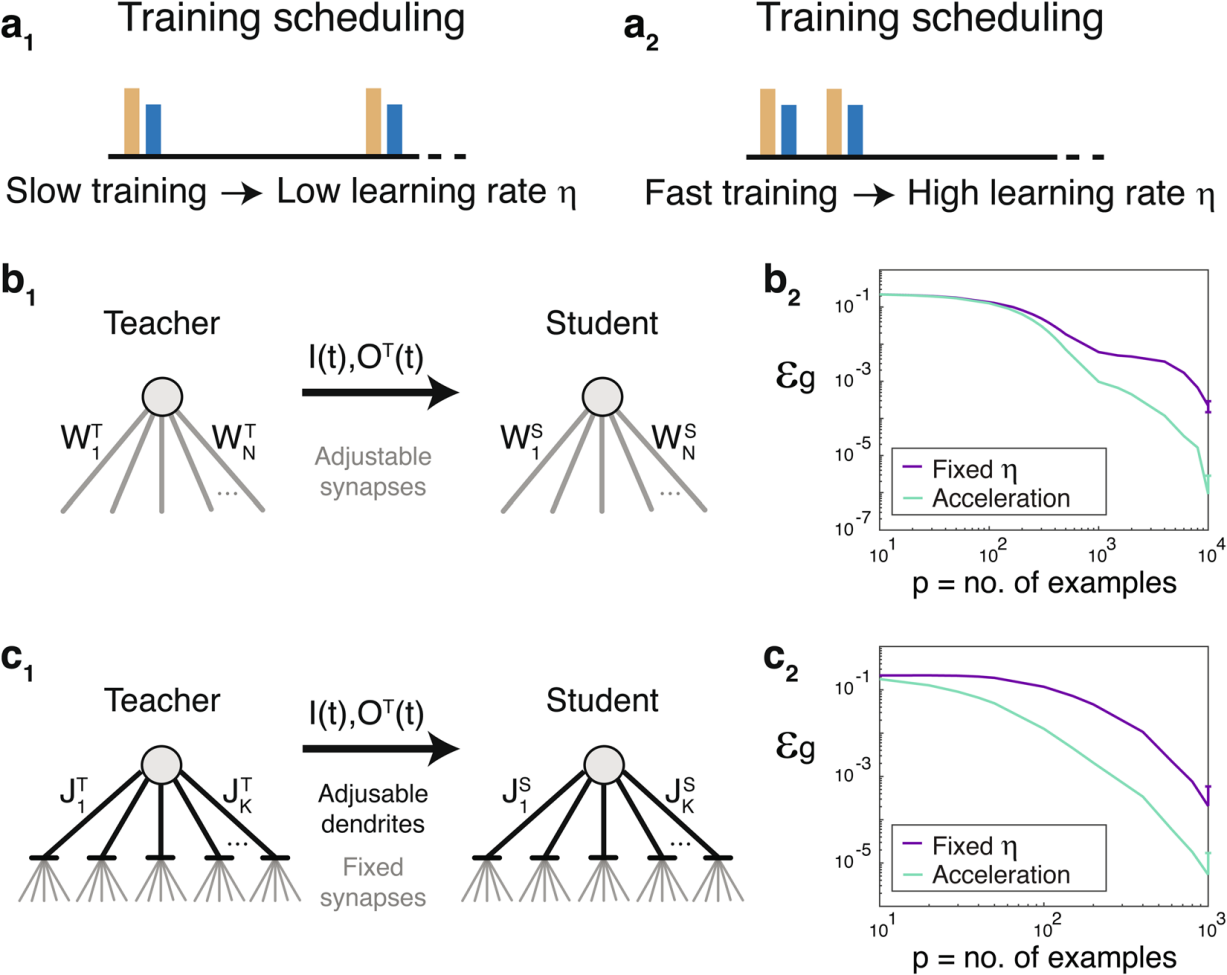
Acceleration of supervised realizable learning rules based on the biologically inspired mechanism. (**a**) The implication of the biological mechanism (Fig. 1) indicating that training scheduling with low/high frequency (**a**_**1**_**/a**_**2**_) results in a low/high learning rate, η. (**b**) Synaptic learning. (**b**_**1**_) A perceptron with $1000$ asynchronous inputs and a leaky integrate-and-fire output unit. The synapses of the teacher/student, W^T^/W^S^, are dynamically updated following a biological adaptation rule, a spike-time-dependent-plasticity (Materials and Methods). (**b**_**2**_) The generalization error, ε_*g*_, for the training of the student in **b**_**1**_ using fixed learning step $\eta =0.001\,$(purple) and for the accelerating scenario ${\eta }^{t+1}={\eta }^{t}\cdot \exp (-0.1)+0.01\cdot sign({O}^{T}-{O}^{S})\,$(green), where O^T^/O^S^ stands for a teacher/student output, and the initial $\eta =0.001.\,$(**c**) Dendritic learning. (**c**_**1**_) Dendritic learning, similar to **b**_**1**_, where the $1000$ synapses are fixed and the 200 dendrites of the teacher/student, J^T^/J^S^, are updated using similarly adaptive rule as in **b** (Materials and Methods). (**c**_**2**_) ε_*g*_ for the trained student, **c**_**1**_, using the same scenarios for $\eta$ as in **b**_**2**_. Results, **b**_**2**_ and **c**_**2**_, are averaged over 30 training datasets and the STD of ε_*g*_ is presented for the maximal presented p.

With the increased training frequency, the adaptation process substantially accelerated (Fig. 2a), potentially implying a time-dependent decaying adaptation step-size:

1 $${\eta }_{adap}^{t+1}={\eta }_{adap}^{t}\cdot {e}^{-\frac{\tau }{{\tau }_{0}}}+\varDelta $$

where the current adaptation step, ${\eta }_{adap}^{t+1}$, is equal to the previous one with a decaying weight, $t$ stands for a discrete time step, ${\tau }_{0}$ is a constant, 1/τ stands for the training frequency, and Δ is a constant representing the incremental effect of the current training step. This type of decay process occurs in many biological scenarios and represents, for instance, the decaying concentration of active material due to diffusion. As a generalization of Eq. (1), incoherent consecutive training steps are also allowed, decreasing the dendritic strength and resulting in a −Δ term in Eq. (1).

Using supervised on-line learning of realizable rules and binary classification (Fig. 2b,c), we first examined the impact of the time-dependent adaptation steps (Eq. 1) on accelerating biological learning processes. The teacher provided the student asynchronous-input and binary-output relations^8^, where both had the same architecture of the simplest classifier, the perceptron^9^, and the output nodes were represented by a leaky integrate-and-fire neuron^10^. Two scenarios were examined: synaptic adaptation and dendritic adaptation (Fig. 2b,c and Materials and Methods). Results clearly indicate that the generalization error, ε_*g*_, of the experimentally-inspired time-dependent η (Eq. 1) substantially outperformed the fixed η scenario (Fig. 2b,c). This accelerated learning stems from the fact that weights in synaptic learning converge to the extreme limits, vanishing or above-threshold weights^7^. Hence, the learning step-sizes in coherent dynamics increased toward these extremes, accelerating the learning scenario (Fig. 2b). Similarly, in the dendritic case (Fig. 2c), weights oscillated and synchronized via repeatedly hitting the boundary values^7^. Hence, a faster decay of ε_*g*_ also resulted from learning step acceleration (Fig. 2c).

**Figure 3:**
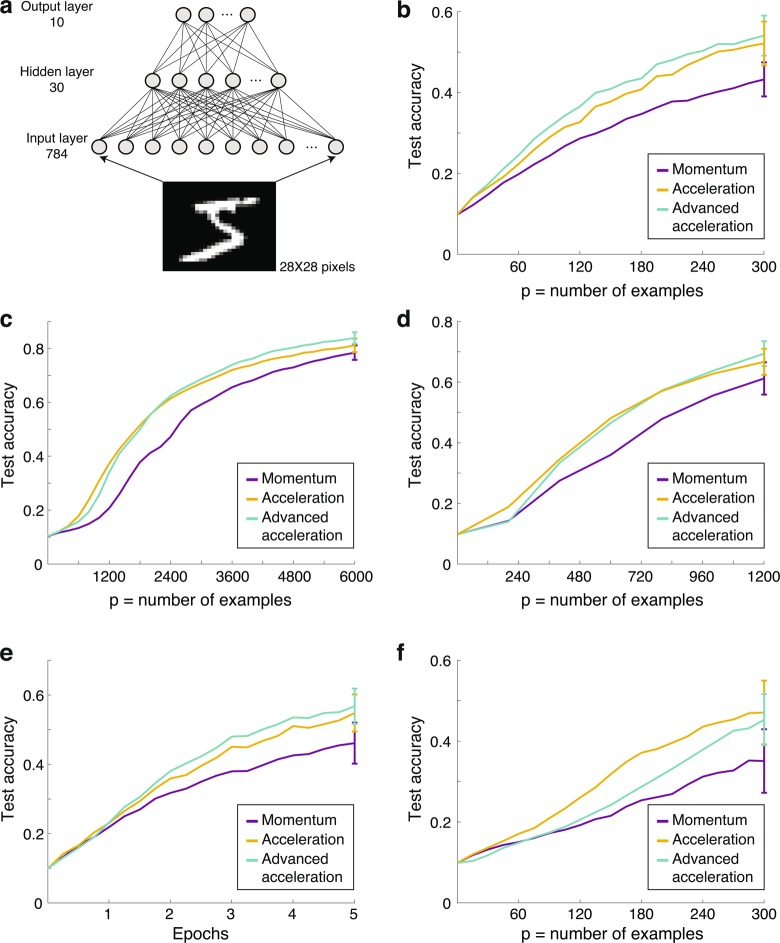
Biological-inspired accelerating learning for the MNIST database in comparison with a common existing learning method. (**a**) The trained network, using backpropagation and cross-entropy cost function, consists of 784 (28 × 28) input units representing the pixels of an MNIST digit, 30 hidden units, and 10 outputs representing the probabilities for the possible labels (Materials and Methods). (**b**) The maximal attainable test accuracy for 300 examples trained only once by the network in **a**, using the following methods; momentum, Eq. (3) (purple), acceleration, Eq. (4) (orange) and advanced acceleration, Eq. (5) (green). (**c**) Similar to **b** using 6000 examples composed of 30 batches of 200. (**d**) Similar to **b** using 1200 examples composed of 24 batches of 50. (**e**) 60 examples are trained 5 times. The total number of presented examples to the network, **a**, is 300 (60 × 5) as in **b**. (**f**) Similar to **b** using mean-square-error cost-function. Results, panels **b-f**, are averaged over 100 training datasets and the STD of the test accuracy is presented for the last trained example.

Next, we examined the experimentally-inspired time-dependent learning step mechanism on the supervised learning of an unrealizable rule using the MNIST database^11^ tested on a neural network. This database consists of a large number of examples of handwritten digits (Fig. 3a) and is commonly used as a prototypical problem for quantifying the generalization performance of machine learning algorithms for various image processing tasks. In this study we use a small subset of the MNIST database without any data extension methods^12–14^. The commonly used trained networks consisted of 784 inputs representing the 28 × 28 pixels of a digit, one hidden layer (30 units in this study), and ten outputs representing the labels (Fig. 3a). The commonly used learning approach is the backpropagation strategy^15^:

2 $${W}^{t+1}={W}^{t}-\eta \cdot {{\nabla }}_{{W}^{t}}C$$

where weight at time-step $t$, ${W}^{t}$, is modified with a step-size η towards the minus sign of the gradient of the cost function, C. An improved approach is the momentum strategy^5,16,17^ and regularization of the weights^18,19^:

3 $${W}^{t+1}=(1-\alpha )\cdot {W}^{t}+{V}^{t+1}$$

$${V}^{t+1}=\mu \cdot {V}^{t}-{\eta }_{0}\cdot {{\nabla }}_{{W}^{t}}C$$

where the momentum, μ, and the regularization, α, are constants in the region [0, 1] and ${\eta }_{0}$ is a constant. We optimized the performance of the momentum strategy (Eq. 3) over $(\mu ,\,\alpha ,\,{\eta }_{0})$ for a limited training dataset using the cross-entropy cost function (Materials and Methods) and compared its performance with the following two experimentally-inspired learning mechanisms consisting of time-dependent η.

In the first approach, acceleration, the time-dependent η, and the update rules for weight are given by these equations:

4 $${W}^{t+1}=(1-\alpha )\cdot {W}^{t}-|{\eta }^{t+1}|\cdot {{\nabla }}_{{W}^{t}}C$$

$${\eta }^{t+1}={\eta }^{t}\cdot {e}^{-\tau }+{A}_{1/2}\cdot tanh({\beta }_{1/2}\cdot {{\nabla }}_{{W}^{t}}C)$$

where τ is the positive decaying factor, ${A}_{1}$ and ${\beta }_{1}$ are constants representing the amplitude and the gain between the input and the hidden layers, respectively, and ${A}_{2}$ and ${\beta }_{2}$ represent the same between the hidden and the output layers. It is evident that coherent consecutive gradients of weight, i.e., with the same sign, increased its conjugate η. Note, in the limit $\beta \to \infty$, the equation for η was simplified, ${\eta }^{t+1}={\eta }^{t}\cdot \exp (-\tau )+A\cdot sign({{\nabla }}_{{{\rm{W}}}^{{\rm{t}}}}C)$. The second approach, advanced acceleration, combines the two previous approaches (Eqs. 3, 4):

5 $${W}^{t+1}=(1-\alpha )\cdot {W}^{t}+{V}^{t+1}$$

$${V}^{t+1}=\mu \cdot {V}^{t}-|{\eta }^{t+1}|\cdot {{\nabla }}_{{W}^{t}}C$$

$${\eta }^{t+1}={\eta }^{t}\cdot {e}^{-\tau }+{A}_{1/2}\cdot tanh({\beta }_{1/2}\cdot {{\nabla }}_{{W}^{t}}C)$$

For the two experimentally-inspired accelerated-approaches (Eqs. 4, 5), the changes in weights and $\eta$ depend on the higher moments of gradients, in contrast with the linear dependence of the momentum approach (Eq. 3). Given a limited subset of the dataset examples the performance of the accelerated approaches was maximized over six (Eq. 4) and seven (Eq. 5) parameters (Materials and Methods).

The on-line training set consisted of 300 randomly chosen examples: each label appeared 30 times within a random order. After 300 learning steps, the accelerated approaches outperformed the momentum method by more than 25%, and the test accuracy increased from about 0.43 to 0.54, respectively (Fig. 3b). Note, the acceleration approach (Eq. 4) showed similar performance to the advanced acceleration approach (Eq. 5). These improved results were found to be robust also for on-line training based on 6000 examples (30 batches of size 200) (Fig. 3c) and 1200 examples (24 batches of size 50) (Fig. 3d). For both cases (Fig. 3c,d), the advanced acceleration approach (Eq. 5) offered the best performance. We repeated the training using the same 60 examples 5 times (60 × 5 = 300 training in total); compared with using 300 examples for training once, the 60 × 5 approach yielded better performance: using the advanced acceleration approach test accuracy increased from 0.54 to 0.57 (Fig. 3e and Supplementary Fig. 2). For a given number of network updates, results demonstrate that smaller example sets yield more information. This result stems from the random training order of a randomly selected small dataset, e.g., 60 or 300 examples, consisting of a balanced appearance for each label. Around equalized trained label appearances, there are more temporal fluctuations for a dataset involving 300 examples with 30 appearances for each label than for one involving 5 sets of 60 examples with 6 appearances for each label. Indeed, for a training set of 300 distinct examples composed of 5 subsets of 60 balanced examples, a test accuracy of 0.57 was achieved (Supplementary Fig. 3), and for one with 30 subsets of 10 examples where each label appears once, the test accuracy increased further to 0.67 and to 0.7 for a fixed label order (Supplementary Fig. 4). Results indicate that in order to maximize the test accuracy for on-line scenarios and especially for small datasets, the balanced set of examples and their balanced temporal training order are important ingredients.

## Conclusions

Based on increased η with coherent consecutive gradients, the brain-inspired accelerated-learning mechanism outperforms existing common ML strategies for small sets of training examples^20^. Consistent results occur across various cost functions, e.g., square cost-function, however, with a relatively diminished performance (Fig. 3f). Because the performance maximization for a given dataset depends on the selected acceleration approach (Fig. 3b,c), adapting the learning approach during the training process may improve performance. Nevertheless, in addition to possible advanced nonlinear functions for updating η, given the number of network updates, the ultimate scheduling of acceleration approaches and the ordering of trained examples to maximize the performance deserves further research. The presented bridge from experimental neuroscience to ML is expected to further advance decision making using limited databases, which is the reality in many aspects of human activity^21^, robotic control^22,23^, and network optimization^24,25^.

## Materials and Methods

### *In-Vitro* experiments

The experimental methods are similar to our previous studies^6,7^ and only the modifications are presented. All procedures were in accordance with the National Institutes of Health Guide for the Care and Use of Laboratory Animals and Bar-Ilan University Guidelines for the Use and Care of Laboratory Animals in Research and were approved and supervised by the Bar-Ilan University Animal Care and Use Committee.

#### Experiments protocol

The details of the experimental protocol of Figure 1 are as follows. The neuronal response latency (NRL) was used to accurately adjust the time-lag between intracellular evoked spikes or EPSPs originated from consecutive intra- and extra- cellular stimulations to be in the range of 1–4 ms. We note that an above-threshold extracellular stimulation given shortly, e.g. 2 ms, after an above-threshold intracellular stimulation, does not result in an evoked spike, and can be used to enhance adaptation. The thresholds and NRL were rechecked at the end of the experiment, in order to ensure their stability.

#### Statistical analysis

The demonstrated results were repeated tens of times on many cultures.

### Simulations of biological neural networks

The simulation methods are similar to our previous studies^8^ and only the used parameters and modifications are presented.

#### Inputs generation

Each input was composed of N/2 randomly stimulated input units. For each stimulated unit a random delay and a stimulation amplitude were chosen from given distributions. The delays were randomly chosen from a uniform distribution with a resolution of 1 (0.001) ms, such that the average time-lag between two consecutive stimulations was 2 (10) ms for the synaptic (dendritic) scenario. Stimulation amplitudes were randomly chosen from a uniform distribution in the range [0.8, 1.2]. Note that the reported results are qualitatively robust to the scenario where all the non-zero amplitudes equal 1. In the dendritic scenario, the five ${W}_{m}$ connected to the same dendrite were stimulated sequentially in a random order and with an average time-lag of 10 ms between consecutive stimulations.

#### Calculating the generalization error

The estimation consisted of up to 20,000 inputs presented to the teacher and the student, where each input generates about 30/200 evoked spikes for the synaptic/dendritic scenario. The generalization error is defined as

$$\begin{array}{c}{\varepsilon }_{g}=\frac{total\,no.of\,mismatch\,firing\,}{total\,no.of\,stimulations}.\end{array}$$

The details of the simulations in Figure 2 are as follows. Panel B: $\{{W}_{m}\}$ were chosen from a uniform distribution in the range [0.1, 0.2]. The adaptation and learning steps were A = 0.05 and ${\rm{\eta }}$ = 1/1000, respectively. ${W}_{m}$ was bounded from above by 1.5 and from below by 10^−4^. The fixed learning rate was compared to the accelerating method using adaptive learning step:

$${\eta }^{t+1}={\eta }^{t}\cdot {e}^{-\tau }+B\cdot sign({O}^{T}-{O}^{S})$$

using $\tau$ = 0.1, B = 0.01 and $\eta$ was initiated as 1/1000.

Panel C: $\{{W}_{m}\}$ were chosen from a uniform distribution in the range [0.1, 0.9] and then were normalized to a mean equals to 0.5. $\{{J}_{i}\}$ were chosen from a uniform distribution in the range [0.5, 1.5]. Stimulations with low amplitudes (0.01) were given to the N/2 unstimulated input units, resulting in non-frozen ${J}_{i}$. The adaptation and learning steps were A = 0.05 and ${\rm{\eta }}$ = 1/1000, respectively. ${J}_{i}$ was bounded from below by 0.1 and from above by 3. The fixed learning rate was compared to the accelerating method using adaptive learning step:

$${\eta }^{t+1}={\eta }^{t}\cdot {e}^{-\tau }+B\cdot sign({O}^{T}-{O}^{S})$$

using $\tau$ = 0.1, B = 0.01 and $\eta$ was initiated as 1/1000.

### Simulations of neural network

#### Architecture

The feedforward neural network contains 784 input units, 30 hidden units and 10 output units in a fully connected architecture. Each unit in the hidden and the output layers has an additional input from a bias unit. Weights from the input layer to the hidden layer, W_1_, and from the hidden layer to the output layer, W_2_, were randomly chosen from a Gaussian distribution with a zero average and standard deviation equals 1. All weights were normalized such that all input weights to each hidden unit have an average equals 0 and a STD equals 1. The initial value of the bias of each weights was set to 1. We trained the network on the handwritten digits dataset, MNIST, using gradient descent. The inputs, examples from the train dataset, contain 784 pixel values in the range [0, 255]. We normalized the inputs such that the average and the STD are equal to 0 and 1, respectively.

#### Forward propagation

The output of a single unit in the hidden layer, ${a}_{j}^{1}$, was calculated as:

$${z}_{j}^{1}=\sum _{i}({W}_{ij}^{1}\cdot {X}_{i})$$

$${a}_{j}^{1}=\frac{1}{1+{e}^{-{Z}_{j}^{1}}}$$

where ${W}_{ij}^{1}$ is the weight from the i^th^ input to the j^th^ hidden unit, ${X}_{i}$ is the i^th^ input, and b^1^_j_ is the bias for the j^th^ hidden unit.

For the output layer, the output of a single unit, ${a}_{j}^{2}$ was calculated as:

$${z}_{j}^{2}=\sum _{i}({W}_{ij}^{2}\cdot {a}_{i}^{1})$$

$${a}_{j}^{2}=\frac{1}{1+{e}^{-{Z}_{j}^{2}}}$$

where ${W}_{ij}^{2}$ is the weight from the i^th^ hidden unit to the j^th^ output unit, ${a}_{i}^{1}$ is the output of the i^th^ unit in the hidden layer, and b^2^_j_ is the bias for the j^th^ output unit.

#### Back propagation

We used two different cost functions; the first was the cross entropy:

$$C=-\frac{1}{N}\sum _{n}[y\ast \,\log (a)+(1-y)\ast \,\log (1-a)]$$

and the second was the mean square error (MSE):

$$C=\frac{1}{2N}\sum _{n}{(y-a)}^{2}$$

where y are the desired labels and $a$ stands for the current 10 output units of the output layer. The summation is over all training examples, N.

The backpropagation method computes the gradient for each weight with respect to the chosen cost function. The weights and biases were updated according to 3 different methods:

1. Momentum
  The weights update:
  $${W}^{t+1}=(1-\alpha )\cdot {W}^{t}+{V}^{t+1}$$
  $${V}^{t+1}=\mu \cdot {V}^{t}-{\eta }_{0}\cdot {\nabla }_{{{\rm{W}}}^{{\rm{t}}}}C$$
  where t is the discrete time-step W are the weights, α is a regularization constant, $\eta$ is the fixed learning rate, and ${\nabla }_{{{\rm{W}}}^{{\rm{t}}}}C$ is the gradient of the cost function for each weight at time t. V was initialized as: $-{\eta }_{0}\cdot {\nabla }_{{\rm{W}}}{C}_{first}$, where ${\nabla }_{W}{C}_{first}$ is the first computed gradient and the biases update:
  $${V}_{b}^{t+1}=\mu \cdot {V}_{b}^{t}-{\eta }_{0}\cdot {\nabla }_{{b}^{t}}C$$
  $${b}^{t+1}={b}^{t}+{V}_{b}^{t+1}$$
  where ${\nabla }_{b}C$ is the gradient of the cost function of each bias with respect to its weight, b, and V_b_ was initialized as: $-\eta \cdot {\nabla }_{{\rm{b}}}{C}_{first}$, where ${\nabla }_{b}{C}_{first}$ is the first computed bias gradient.
2. Acceleration
  The weights update:
  $${W}^{t+1}=(1-\alpha )\cdot {W}^{t}-|{\eta }^{t+1}|\cdot {\nabla }_{{W}^{t}}C$$
  $${\eta }^{t+1}={\eta }^{t}\cdot {e}^{-\tau }+{A}_{1/2}\cdot tanh({\beta }_{1/2}\cdot {\nabla }_{{W}^{t}}C)$$
  where $\eta$ is defined for each weight, $\,{A}_{1}$ and ${\beta }_{1}$ are constants representing the amplitude and the gain between the input and the hidden layers, respectively, and ${A}_{2}$ and ${\beta }_{2}$ represent the same between the hidden and the output layers. $\eta$ was initialized as: ${A}_{1/2}\cdot tanh({\beta }_{1/2}\cdot {\nabla }_{W}{C}_{first})$, where ${\nabla }_{W}{C}_{first}$ is the first computed gradient and the biases update:
  $${b}^{t+1}={b}^{t}-|{\eta }_{b}^{t+1}|\cdot {\nabla }_{{b}^{t}}C$$
  $${\eta }_{b}^{t+1}={\eta }_{b}^{t}\cdot {e}^{-\tau }+{A}_{1/2}\cdot tanh({\beta }_{1/2}\cdot {\nabla }_{{b}^{t}}C)$$
3. Advanced acceleration:

        The weights update

$${W}^{t+1}=(1-\alpha )\cdot {W}^{t}+{V}^{t+1}$$

$${V}^{t+1}=\mu \cdot {V}^{t}-{\eta }^{t+1}\cdot {\nabla }_{{W}^{t}}C$$

$${\eta }^{t+1}={\eta }^{t}\cdot {e}^{-\tau }+{A}_{1/2}\cdot tanh({\beta }_{1/2}\cdot {\nabla }_{{W}^{t}}C)$$

        and the biases update:

$${b}^{t+1}={b}^{t}+{V}_{b}^{t+1}$$

$${V}_{b}^{t+1}=\mu \cdot {V}_{b}^{t}-{\eta }_{b}^{t+1}\cdot {\nabla }_{{b}^{t}}C$$

$${\eta }_{b}^{t+1}={\eta }_{b}^{t}\cdot {e}^{-\tau }+{A}_{1/2}\cdot tanh({\beta }_{1/2}\cdot {\nabla }_{{b}^{t}}C)$$

#### Testing the network

The network classification accuracy was tested on the MNIST dataset for testing, containing 10,000 inputs. The test inputs were also normalized to have an average of each equals to 0 and a STD equals to 1.

#### Optimization

For each update method the parameters were chosen to maximize the test accuracy. For optimization we first used a grid of the adjustable parameters followed by a fine tuning with higher resolution for each parameter. The optimization was performed over 3 parameters for the momentum method ($\mu ,{\eta }_{0},\alpha$), 6 parameters for the acceleration method (${A}_{1},{A}_{2},{\beta }_{1},{\beta }_{2},\tau ,\,\alpha$) and for 7 parameters for the advanced acceleration method (${A}_{1},{A}_{2},{\beta }_{1},{\beta }_{2},\tau ,\,\alpha ,\mu$).

The details of the simulations in Figure 3 are as follows. Panel B: The feed forward neural network was presented with 300 examples with equally number of appearance of each digit. The cross entropy cost function was used and the following parameters for each method: momentum with $\mu =0.9,{\eta }_{0}=0.02,\alpha =0$, acceleration with ${A}_{1}=0.1,{A}_{2}=0.27,{\beta }_{1}=\,5000,{\beta }_{2}=\,900,\tau =\,0.598,\,\alpha =0.003$, and advanced acceleration with ${A}_{1}=0.07,{A}_{2}=0.07,{\beta }_{1}=\infty ,{\beta }_{2}=\,\infty ,\tau =0.29,\,\alpha =\,0.005,\mu =0.5$. Results are presented as the average of 100 different runs, and typical error bars are presented for the last point.

Panel C: The feed forward neural network was presented with 6000 examples randomly taken from the 60000 examples in the training dataset, and presented to the network with 30 mini-batches of size 200. The cross entropy cost function was used and the following parameters for each method: momentum with $$\mu =0.68,{\eta }_{0}=0.65,\alpha =0.08$$, acceleration with $${A}_{1}=3,{A}_{2}=0.7,{\beta }_{1}=\,1500,{\beta }_{2}=\,1000,\tau =\,0.4,$$$$\alpha =0.065$$, and advanced acceleration with $${A}_{1}=0.5,{A}_{2}=0.5,{\beta }_{1}=\infty ,{\beta }_{2}=\,\infty ,\tau =0.1,\,\alpha =\,0.1,$$$$\mu =0.55$$. Results are presented as the average of 100 different runs, and typical error bars are presented for the last point.

Panel D: The feed forward neural network was presented with 1200 examples randomly taken from the 60000 examples in the training dataset, and presented to the network with 24 mini-batches of size 50. The cross entropy cost function was used and the following parameters for each method: momentum with $$\mu =0.75,$$$${\eta }_{0}=0.6,\alpha =0.11$$, acceleration with $${A}_{1}=1.15,{A}_{2}=0.6,{\beta }_{1}=\,4500,{\beta }_{2}=\,3500,\tau =\,0.1,\,\alpha =0.055$$, and advanced acceleration with $${A}_{1}=0.55,{A}_{2}=0.55,{\beta }_{1}=\infty ,{\beta }_{2}=\,\infty ,\tau =0.1,\,\alpha =\,0.1,\mu =0.55$$. Results are presented as the average of 100 different runs, and typical error bars are presented for the last point.

Panel E: The feed forward neural network was presented with 60 with equally number of appearance of each digit. The 60 examples were presented to the network 5 times. The cross entropy cost function was used and the following parameters for each method: momentum with $\mu =0.87,{\eta }_{0}=0.035,\alpha =0.005$, acceleration with ${A}_{1}=0.11,{A}_{2}=0.26,{\beta }_{1}=\,2000,{\beta }_{2}=\,2500,\tau =\,0.45,\,\alpha =0.008$, and advanced acceleration with ${A}_{1}=0.035,{A}_{2}=0.02,{\beta }_{1}=4500,{\beta }_{2}=\,5,\tau =0.0619,\alpha =\,0.01,\mu =0.6$. Results are presented as the average of 100 different runs, and typical error bars are presented for the last point.

Panel F: The feed forward neural network was presented with 300 examples with equally number of appearance of each digit. The mean-square-error cost function was used and the following parameters for each method: momentum with $$\mu =0.6,{\eta }_{0}=0.35,\alpha =0.005$$, acceleration with $${A}_{1}=0.95,{A}_{2}=0.25,{\beta }_{1}=\,5000,$$$${\beta }_{2}=\,40,\tau =\,0.15,\alpha =0.008$$, and advanced acceleration with $${A}_{1}=0.06,{A}_{2}=0.09,{\beta }_{1}=2100,{\beta }_{2}=\,1,$$$$\tau =0.015,\,\alpha =\,0.005,\mu =0.8$$. Results are presented as the average of 100 different runs, and typical error bars are presented for the last point.

The feed forward neural network was presented with 300 examples with equally number of appearance of each digit. The examples are composed of 5 subsets of 60 balanced examples, where in every subset each label appears exactly 6 times. The cross entropy cost function was used and the following parameters for each method: momentum with $\mu =0.87,{\eta }_{0}=0.035,\alpha =0.005$, acceleration with ${A}_{1}=0.11,{A}_{2}=0.26,{\beta }_{1}=\,2000,{\beta }_{2}=\,2500,\tau =\,0.45,\,\alpha =0.008$, and advanced acceleration with ${A}_{1}=0.035,{A}_{2}=0.02,{\beta }_{1}=4500,{\beta }_{2}=\,5,\tau =0.0619,\,\alpha =\,0.01,\mu =0.6$. Results are presented as the average of 100 different runs, and typical error bars are presented for the last point.

The feedforward neural network was presented with 300 examples with equally number of appearance of each digit. The examples are composed of 30 subsets of 10 balanced examples, where in every subset each label appears exactly one time. Results are also presented for the advanced acceleration method where for each subset of 10 examples, the labels were in a fixed order. The cross entropy cost function was used and the following parameters for each method: momentum with $\mu =0.87,{\eta }_{0}=0.035,\alpha =0.005$, acceleration with ${A}_{1}=0.11,{A}_{2}=0.26,{\beta }_{1}=\,2000,{\beta }_{2}=\,2500,\tau =\,0.45,\,\alpha =0.008$, and advanced acceleration with ${A}_{1}=0.035,{A}_{2}=0.02,{\beta }_{1}=4500,{\beta }_{2}=\,5,\tau =0.0619,\alpha =\,0.01,\mu =0.6$. Results are presented as the average of 100 different runs, and typical error bars are presented for the last point.

## Supplementary information

Supplementary Information.
