# Publisher Correction: Brain experiments imply adaptation mechanisms which outperform common AI learning algorithms

**DOI:** 10.1038/s41598-020-65969-z

**Published:** 2020-06-04

**Authors:** Shira Sardi, Roni Vardi, Yuval Meir, Yael Tugendhaft, Shiri Hodassman, Amir Goldental, Ido Kanter

**Affiliations:** 1 Department of Physics, Bar-Ilan University, Ramat-Gan, 52900 Israel; 2 Gonda Interdisciplinary Brain Research Center and the Goodman Faculty of Life Sciences, Bar-Ilan University, Ramat-Gan, 52900 Israel

Correction to: *Scientific Reports* 10.1038/s41598-020-63755-5, published online 23 April 2020

This Article contains typographical errors in the Forward Propagation equation, where

$${z}_{j}^{1}=\sum _{i}({W}_{ij}^{1}\cdot {X}_{i})$$

should read:

$${z}_{j}^{1}=\sum _{i}({W}_{ij}^{1}\cdot {X}_{i})+{b}_{j}^{1}$$

and

$${z}_{j}^{2}=\sum _{i}({W}_{ij}^{2}\cdot {a}_{i}^{1})$$

should read:

$${z}_{j}^{2}=\sum _{i}({W}_{ij}^{2}\cdot {a}_{i}^{1})+{b}_{j}^{2}$$

