# Supplementary material for: Brain experiments imply adaptation mechanisms which outperform common AI learning algorithms
Source: Sci Rep. 2020 Apr 23;10:6923. doi: 10.1038/s41598-020-63755-5 (PMC7181840; doi:10.1038/s41598-020-63755-5)
Supplement: Supplementary file 1 — Supplementary Information. [file 41598_2020_63755_MOESM1_ESM.pdf]

Supplementary Information

Brain-experiments imply adaptation mechanisms which outperform common AI learning algorithms

Shira Sardi[1], Roni Vardi[2], Yuval Meir[1], Yael Tugendhaft[1], Shiri Hodassman[1], Amir Goldental[1] & Ido Kanter[1,2]
[1]Department of Physics, Bar-Ilan University, Ramat-Gan, 52900, Israel.
[2]Gonda Interdisciplinary Brain Research Center and the Goodman Faculty of Life Sciences, Bar-Ilan University, Ramat-Gan, 52900, Israel.

Correspondence to: ido.kanter@biu.ac.il

Supplementary Figures

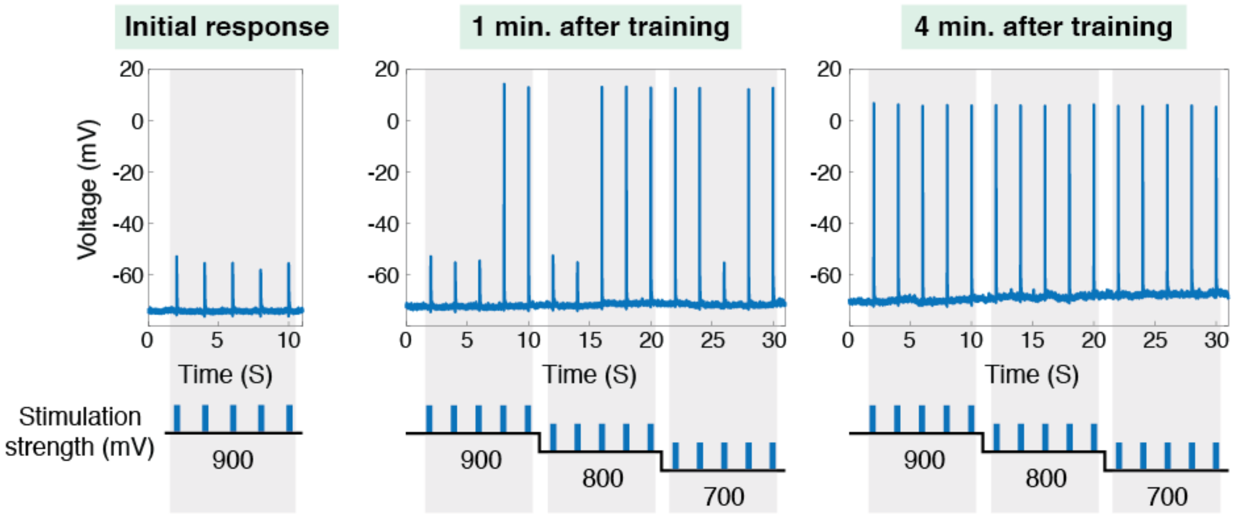

Supplementary Figure 1. The effect of adaptation after one minute is further enhanced after additional several minutes.

An example of the first type of experiments, as in Fig. 1c, where decreasing extracellular stimulation amplitude is used to estimate the threshold using intracellular recording (left), and enhanced responses measured one minute after the termination of the training, (middle). Further enhancement in the response is measured 4 minutes after termination of the training (right).

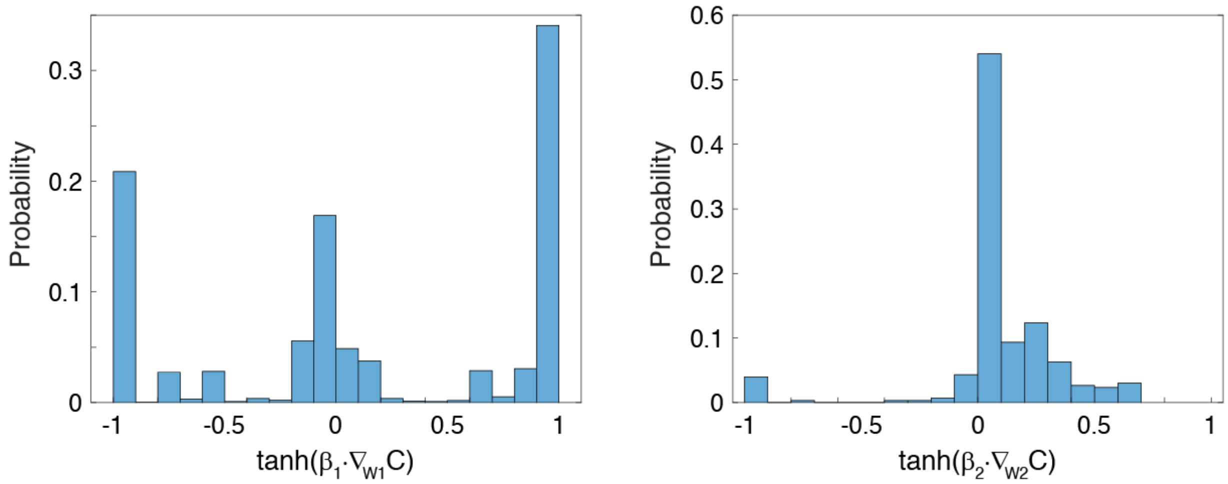

Supplementary Figure 2. The maximal test accuracy for the advanced acceleration method is achieved in the finite $\boldsymbol{\beta}$ limit.

Left: The probability distribution for $\tanh(\beta_1 \cdot \nabla_{W_1} C)$, taken from the training of last example in Fig. 3e. Right: The probability distribution for $\tanh(\beta_2 \cdot \nabla_{W_2} C)$, taken from the training of last example in Fig. 3e. Both histograms indicate that the maximal test accuracy is obtained using finite β, i.e. $|\tanh\left(\beta_{1/2} \cdot \nabla_{W_{1/2}} C\right)| < 1$ with finite probability, which cannot be simplified to $sign\left(\nabla_{w_{1/2}} C\right)$.

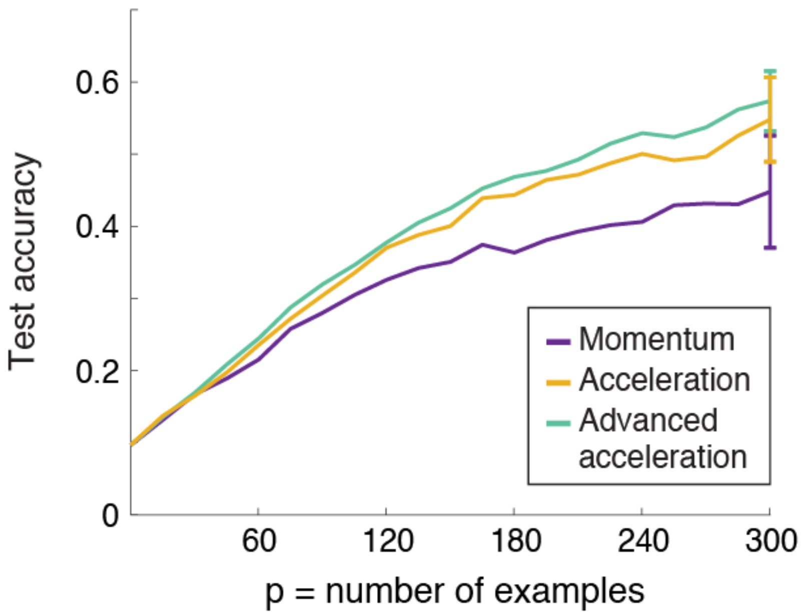

Supplementary Figure 3. Maximal test accuracy for training set composed of 5 balanced subsets of 60 examples.

The maximal attainable test accuracy for 300 examples trained only once by the network in Fig. 3a. The training set of 300 distinct examples was composed of 5 subsets of 60 balanced examples, i.e. each one of the labels appears 6 times, using the following methods; momentum, eq. (3) (purple), acceleration, eq. (4) (orange) and advanced acceleration, eq. (5) (green), and the test accuracy is ~0.57. The parameters are given in the Methods and Materials section. Results were averaged over 100 training sets and the STD is presented for p=300.

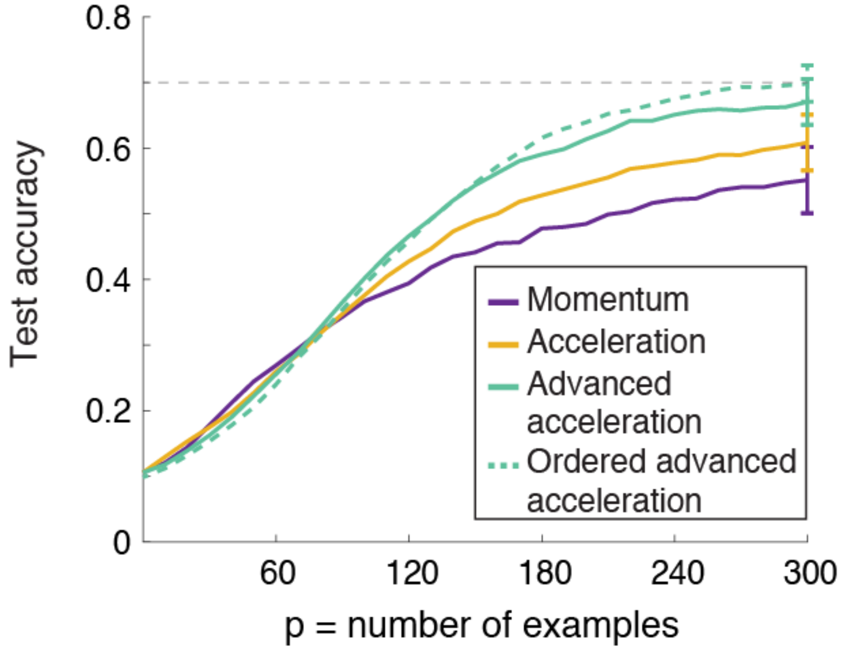

Supplementary Figure 4. Maximal test accuracy for a training set composed of 30 subsets of 10 examples, where each label appears once.

The maximal attainable test accuracy for 300 examples trained only once by the network in Fig. 3a. The training set of 300 distinct examples was composed of 30 subsets of 10 examples, where each label appears once, using the following methods; momentum, eq. (3) (purple), acceleration, eq. (4) (orange) and advanced acceleration, eq. (5) (green), and the test accuracy is ~0.67. The advanced acceleration with a fixed order of labels within all 30 subsets of 10 examples results in a test accuracy of ~0.7 (dashed green). The parameters are given in the Methods and Materials section. Results were averaged over 100 training sets and the STD is presented for p=300.
